# Northern lights assay: a versatile method for comprehensive detection of DNA damage

**DOI:** 10.1093/nar/gky645

**Published:** 2018-07-24

**Authors:** Bjarki Gudmundsson, Hans G Thormar, Albert Sigurdsson, Wendy Dankers, Margret Steinarsdottir, Stefan Hermanowicz, Stefan Sigurdsson, David Olafsson, Anna M Halldorsdottir, Stephen Meyn, Jon J Jonsson

**Affiliations:** 1 Department of Biochemistry and Molecular Biology, University of Iceland, Reykjavik IS-101, Iceland; 2 Department of Genetics and Molecular Medicine, Landspitali–National University Hospital, Reykjavik IS-101, Iceland; 3 Lifeind ehf., Reykjavik IS-101, Iceland; 4 Faculty of Medicine, University of Iceland, Reykjavik IS-101, Iceland; 5 The Blood Bank, Landspitali–National University Hospital, Reykjavik IS-101, Iceland; 6 Department of Paediatrics, The Hospital for Sick Children, Toronto, ON, M5G 1X8, Canada; 7 University of Toronto, Toronto, ON, M5S 1A8, Canada; 8 Center for Human Genomics and Precision Medicine, School of Medicine and Public Health, University of Wisconsin, Madison, Wisconsin 53705, USA

## Abstract

DNA damage assays have various limitations in types of lesions detected, sensitivity, specificity and samples that can be analyzed. The Northern Lights Assay (NLA) is based on 2D Strandness-Dependent Electrophoresis (2D-SDE), a technique that separates nucleic acids based on length, strandness, structure and conformation changes induced by damage. NLA is run on a microgel platform in 20–25 min. Each specimen is analyzed in pairs of non-digested DNA to detect single- and double-stranded breaks (DSBs) and Mbo I-digested DNA to detect other lesions. We used NLA to evaluate DNA in solution and isolated from human cells treated with various genotoxic agents. NLA detected and distinguished between single- and DSBs, interstrand and intrastrand DNA crosslinks, and denatured single-stranded DNA. NLA was sufficiently sensitive to detect biologically relevant amount of DNA damage. NLA is a versatile, sensitive and simple method for comprehensive and simultaneous analysis of multiple types of damage, both in purified DNA and in DNA isolated from cells and body fluids. NLA can be used to evaluate DNA quality in biosamples, monitor complex molecular procedures, assess genotoxicity, diagnose genome instability, facilitate cancer theranostics and in basic nucleic acids research.

## INTRODUCTION

Cells are constantly exposed to genotoxic stress arising either from cell metabolism or external genotoxic agents. The damage that arises from genotoxic stress, including single-stranded breaks (SSB), double-stranded breaks (DSB), crosslinks and bulky adducts, can lead to mutagenesis or cell death if not repaired. Consequently, DNA damage assessment plays an important role in many areas of basic biomedical science and clinical medicine. It also has multiple applications in quality assessment of biosamples and in complex molecular procedures.

Detection of different types of DNA damage is important for diagnosis of genetic diseases associated with aberrant DNA repair. Examples include defects in crosslink repair in patients with Fanconi anemia (FA) (1), UV-induced damage repair in xeroderma pigmentosum patients (2) and ionizing radiation-induced DSB repair in ataxia telangiectasia patients (3). The efficacy of many clinically important cancer chemotherapeutic agents is thought to depend on their capacity to form DNA damage (4–6). Detection of the DNA damage induced by these agents can be used to estimate their therapeutic efficacy or side effects. Examples are the crosslinking agents cisplatin, used to treat ovarian, testicular and cervical cancers; carboplatin, used mainly for ovarian cancer; mitomycin C (MMC), used to treat oesophageal and bladder cancer; and melphalan, a nitrogen mustard derivative used to treat multiple myeloma (7). Other chemotherapeutic agents lead to formation of DNA breaks (8–10). These include PARP inhibitors (10) which are in clinical trials as chemotherapeutic agents to treat BRCA-deficient breast cancer, and topoisomerase inhibitors such as camptothecin, doxorubicin and etoposide (6,11). In addition to causing DNA breaks, etoposide also induces apoptosis (12) which is characterized by the formation of nucleosomal-sized DNA fragments.

Currently, there are several methods for detecting DNA damage and repair *in vitro* and in biological systems. The comet assay is a versatile technique to detect DNA damage at the single cell level (13). Originally developed to detect DNA breaks (13,14), it was subsequently modified to detect other types of DNA damage (15), including interstrand DNA crosslinks, UV-damage and oxidative damage. The alkaline elution assay (16) detects SSB in cells by measuring the rate of DNA elution from cellulose filter membranes. The fluorometric alkaline DNA unwinding assay (FADU assay) is based on partial denaturation of double-stranded DNA (dsDNA) under controlled alkaline conditions where breaks serve as starting positions of unwinding during the alkaline denaturation of chromosomal DNA in a cell lysate (17). In addition, DNA breaks and degradation during apoptosis can be detected *in situ* with the Terminal deoxynucleotidyl transferase (TdT) dUTP Nick-End Labeling (TUNEL) assay (18). The current methods for DNA damage detection; (i) are complex or slow to perform, (ii) do not detect many types of DNA damage simultaneously, (iii) are not applicable for DNA in solution or cell-free DNA from body fluids or (iv) require expensive specialized equipment and expertise. A simple method that can detect and distinguish between all major structural DNA lesions in a quick and easy manner in both cellular DNA and cell-free DNA would be a substantial improvement over existing techniques.

The Northern Lights Assay (NLA) for detection of DNA damage is based 2D Strandness-Dependent Electrophoresis (2D-SDE), a method used to assess quality of complex nucleic acids samples and monitor efficiency of molecular procedures (19–22). During the electrophoresis in the first dimension, molecules in the sample are separated based on their length, strandness and damage-induced conformation. Before the second dimension electrophoresis the DNA in the gel is heat-denatured and all molecules become single-stranded. They therefore separate only according to length in the second dimension electrophoresis, unless they contain interstrand crosslinks. In NLA, pairs of samples, one not digested and the other digested with the restriction enzyme Mbo I are analyzed. Mbo I is a four base cutter that digests both single-stranded DNA (ssDNA) and dsDNA (23).

**Figure 1.**
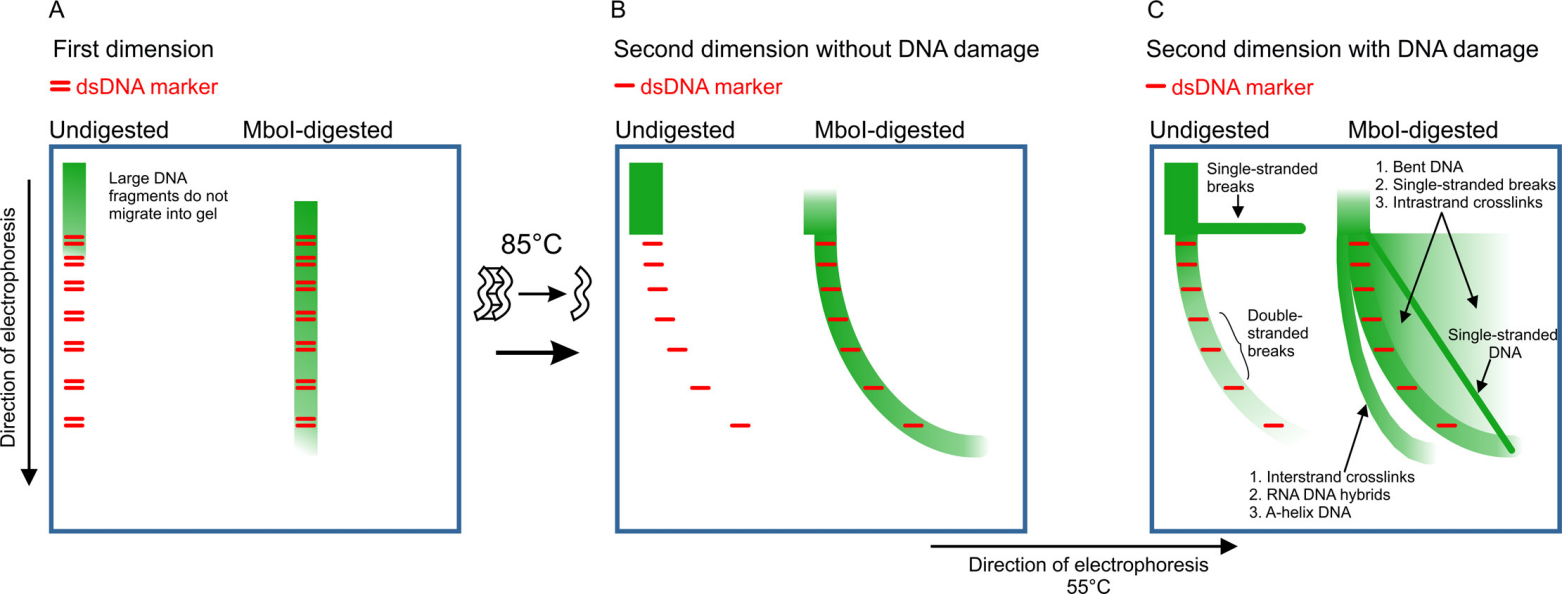
Predicted detection of different types of DNA damage with the NLA. (**A**) Undigested and Mbo I-digested DNA samples being tested are shown in green after first dimension electrophoresis. (**B**) Results for a specimen with intact undamaged DNA. The undigested DNA sample does not migrate into the gel efficiently and the digested sample co-migrates with dsDNA marker (red). (**C**) Results for a specimen with damaged DNA where various lesions can be detected. In the undigested sample, SSB are seen as a horizontal streak extending from the DNA that is too large to migrate into the gel and DSB generate dsDNA molecules that are small enough to co-migrate with the dsDNA marker. Many different DNA lesions can be detected in the Mbo I-digested sample. DNA strands with interstand crosslinks remain covalently attached when denatured. They are larger and migrate slower in the second dimension than ssDNA from originally intact dsDNA. They are therefore placed behind the arc of intact dsDNA molecules in the original sample. DNA molecules that are bent due to monoadducts, instrastrand crosslinks, mismatches or thymidine dimers (shade), and ssDNA (diagonal line) migrate relatively slowly compared to intact dsDNA in the first dimension, but that DNA has normal migration velocity in the second dimension. These molecules are therefore placed in front of normal dsDNA. DNA containing ssDNA breaks gives rise to small fragments on the nicked strand after denaturation. After 2D electrophoresis the smaller fragments will be placed in front of normal dsDNA because of their small size compared to the complementary undamaged strand.

We designed NLA as a versatile method to detect multiple kinds of DNA damage based on the known effects of lesions on the molecular structure and migration characteristics of DNA molecules in 2D-SDE (Figure 1 and Supplementary Figure S7). In this study we evaluated the ability of NLA to detect various types of structural DNA damage, including interstrand and intrastrand DNA crosslinks, SSB and DSB in addition to apoptotic degradation. We tested the method both on DNAs damaged in solution and DNA damaged in living cells. Our results confirm that NLA is a simple sensitive assay for simultaneously detecting various types of DNA damage.

## MATERIALS AND METHODS

### Cisplatin treatment of DNA in solution

Genomic DNA was isolated from blood with Gentra Puregene Blood kit (Qiagen, cat no. 158389) according to the manufacturer’s instructions. DNA (7.5 μg) was digested with 3 U/μg DNA Mbo I (10 U/μl, Thermo Scientific, cat no. ER0812) at 37°C for 1 h. The DNA was purified with Amicon Ultra 30K centrifugal filters (Millipore, cat no. UFC503024) and divided into 1 μg aliquots. Treatment of DNA with cisplatin (cis-Diamminedichloroplatinum(II)) was done according to Murray *et al*. (24). In a total volume of 100 μl, 1 μg DNA was incubated in 25 mM Tris–HCl, pH 7.8 with 1–15 μM of cisplatin (Sigma-Aldrich, cat no. 479306), at 37°C for 18 h in the dark. The DNA was ethanol precipitated with sodium acetate and resuspended in 3 μl TE buffer (10 mM Tris–HCl, 1 mM ethylenediaminetetraacetic acid (EDTA), pH 8.0). For denaturation experiments, 1 μg human genomic DNA, in 3 μl TE, was heated up at 94°C for 4 min. Plasmid pUC 18 was digested with Hinf I (Thermo Scientific, cat no. ER0801) and Ssp I (Thermo Scientific, cat no. ER0771) 1.5 U enzyme per microgram DNA, prior to cisplatin treatment.

### Induction of nicking in DNA in solution

Nicks were formed in 1 μg DNA using either a range of 0.1–1 U Nt.BstNB I (NEB, cat no. R0607S) or 0.008–10 U Nt.CviPII (NEB, cat no. R0626S) in buffer NEB 3 at 55°C for 1 h in total volume of 50 μl.

### Cy5-labeling of DNA ladder

GeneRuler 100 bp Plus ladder (Thermo Scientific, SM0321), ranging in size from 100 to 3000 bp was labeled with fill-in reaction, comprising 5 μg DNA, 5 U T4 polymerase (Thermo Scientific, cat no. EP0061), 1 × T4 polymerase buffer (Thermo Scientific, 67 mM Tris–HCl (pH 8.8), 6.6 mM MgCl_2_, 1 mM dithiothreitol (DTT), 16.5 mM (NH_4_)_2_SO_4_) for 1 min at 37°C to form overhangs. Unlabeled dATP, dGTP and dTTP, and Cy5-labeled dCTP were then added for a final concentration of 100 μM each nucleotide, and fill-in reaction done at 37°C for 15 min and quenched with 1 μl of 0.5 M EDTA. The Cy5-labeled marker was purified with QIAquick PCR purification kit (Qiagen, cat no. 28104) according to manufacturer’s instructions.

### Cell cultures and treatment

Fibroblast cell line GM3440, normal with regard to repair of DNA crosslinks, was obtained from Coriell Institute for Medical Research (cat id. GM03440) (http://www.coriell.org/). BJ foreskin fibroblasts, normal with regard to DNA crosslink repair, were obtained from ATCC (cat no. CRL-2522). *FANCA*^−/−^ cell line complemented with an empty vector (*FANCA*^−/−^ +Vector) (25,26), or with the *FANCA* gene (*FANCA*^−/−^ +WT-*FANCA*) (25), and *FANCD1*^−/−^ cell line RA3226 fibroblasts (27) had been immortalized using a catalytic subunit of telomerase (hTERT) and transformed using HPVE6 and E7 proteins. The sensitivity to treatment with crosslinking agents of cell lines BJ, *FANCA*^−/−^ +Vector, *FANCA*^−/−^ +WT-*FANCA*, and *FANCD1*^−/−^ was characterized in refs. (25–27). Monolayers of cells were cultured at 37°C in a humidified atmosphere of 5% CO_2_ in Dulbecco’s modified Eagle’s medium (DMEM) (Gibco) supplemented with 2 mM glutamine, 100 IU/ml penicillin, 100 IU/ml streptomycin and 10 or 15% fetal bovine serum (FBS) (Gibco). Cell cultures (1–2 × 10^6^ cells) were treated with a medium containing crosslinking agent for 4–24 h. Cisplatin (Sigma-Aldrich) was added to medium without FBS. MMC (Sigma-Aldrich, cat no. M4287), DEB (Sigma-Aldrich, cat no. 202533) or melphalan (Sigma-Aldrich, cat no. M2011) were added to a complete medium.

For repair experiments, the medium containing the crosslinking agent was removed, the cells were washed twice with 1 × PBS (phosphate-buffered saline) at 37°C and allowed to recover in medium without a crosslinking agent for 24–120 h.

Human umbilical vein endothelial cells (HUVEC) were kindly provided by Dr Gudrun Valdimarsdottir (Department of Biochemistry and Molecular Biology, University of Iceland). The HUVEC primary cells were grown in EGM-2 medium (Lonza, cat no. CC-3162) to confluency, at passage 4. Cells were treated with 5 and 50 μM etoposide (corresponding to 2.9 and 29 μg/ml, respectively) and grown in EGM-2 medium for 48 h. For serum-depletion, the cells were grown in FBS-free DMEM for 4 days and control cells were cultured in DMEM with 1% FBS for 4 days.

Monolayers of MCF-7 (ATCC), luminal breast cancer cell line, normal with regards to *BRCA1* and *BRCA2*, and epithelial pancreatic adenocarcinoma cell line Capan-1 (*BRCA2^−/−^*) (ATCC) were cultured in DMEM supplemented with 20% FBS. Human breast epithelial cell line A176 (*BRCA2*^−/+^) (28) was harvested in H14 medium (DMEM with 250 ng/ml insulin, 10 μg/ml transferrin, 2.6 ng/ml sodium selenite, 10-10 M estradiol, 1.4 × 10-6 M hydrocortisone, 5 μg/ml prolactin). The cell cultures were treated with 5 μM olaparib (AZD-2281, Sigma-Aldrich, cat no SML1858) for 48 h.

The cell cultures were detached with 0.05% trypsin (Gibco) and genomic DNA isolated with Gentra Puregene Cell Kit (Qiagen) protocol for cultured cells according to the manufacturer’s instructions. After isolation, DNA was resuspended in TLE buffer (10 mM Tris–HCl, 0.1 mM EDTA, pH 8.0).

### Cytogenetic analysis

Actively dividing cell cultures were treated with 50 nM MMC for 24 h. The cells were arrested by adding colcemid (0.167 μg/ml final concentration) to the culture media for 2 h, followed by collection with trypsin and centrifugation, hypotonic shock with 0.075 M KCl, and fixation in freshly made 3:1 absolute methanol:glacial acetic acid. Cell specimen suspension (3–4 drops) was placed on microscopic slides and stained with 1:3 Leishman dye: PBS (pH 7.0) mixture and analyzed under the microscope.

### Two-dimensional strandness-dependent electrophoresis (2D-SDE)

2D-SDE (19) was carried out on both minigel and microgel platforms. 2D-SDE on the minigel platform was done as described by Gunnarsson *et al*. (20). Polyacrylamide gels (4%) were made from 30% 29:1 acrylamide:bisacrylamide mixture (Amersham Biosciences) in 1 × tris-borate-EDTA (TBE) and 7 M urea. Mbo I-digested DNA (1 μg) containing 20 ng Cy5-labeled ladder was loaded on the gel in 5% glycerol loading buffer. First dimension electrophoresis, was carried out in a vertical Mini-Protean II system (Bio-Rad) at room temperature in 1 × TBE and constant 20 mA for 20 min. After the first dimension electrophoresis the gel was heated up to 85°C on a dry heat-block for 2 min. Second dimension electrophoresis was carried out perpendicular to the first dimension in 1 × TBE at constant 5 W at 55°C for 14 min in a Multiphor system (Amersham Biosciences) using paper electrode wicks. NLA on a microgel platform was carried out on 4% polyacrylamide gels containing 1.25 × TBE and 7 M urea according to Thormar *et al*. (29). In the first dimension, gel electrophoresis was carried out at 4°C at constant 30 mA for 11 min. Between dimensions, the gel was heated up to 85°C for 2 min. The second dimension electrophoresis was done at 55°C at constant 36 mA for 7 min. Gels were stained post-run in 100 ml 1 × TBE buffer with 1 × ribogreen (Invitrogen—Molecluar Probes, cat no. R11491) and analyzed using the fluorescence-scanning mode of Typhoon 8610 variable mode imager (Amersham Biosciences).

### Quantification of DNA damage

Fractions of intact dsDNA and damaged DNA were quantified using ImageQuant 5.1 TL Toolbox v8.1 software (GE Healthcare Life Sciences). Undigested DNA that did not migrate efficiently into the gel was defined from the well down to the upper edge of the 3000 bp band of the Cy5 ladder (purple in Supplementary Figure S1B and C). The Cy5-labeled ladder was used to define the region of dsDNA that did not overlap with damaged DNA, covering the area flanked by the lower edge of the 100 bp ladder band and upper edge of the 1000 bp band, with width defined by the edges of the marker bands (green in Supplementary Figure S1B and C). Displacement of that area immediately to the left was used to define the area DNA migrating behind the dsDNA (blue in Supplementary Figure S1C). The area immediately to the right of the dsDNA ladder was defined vertically from the edge of the 100 bp ladder band perpendicular up to the edge of the horizontal to the 2000 bp band (red in Supplementary Figure S1C). This area included DNA molecules with slow migration velocity compared to dsDNA in the first dimension electrophoresis, including ssDNA and bent molecules. SSB were measured by defining an area where a streak extending from the large molecules that were too large to migrate into the gel and extending to a point vertically over the 100 bp band in the ladder (brown in Supplementary Figure S1C). Quantification was a relative measurement of density within each gel area, given in percentage. Background correction was achieved by selecting a DNA-free area in the gel (Supplementary Figure S1C), subtracting the density of that area from each DNA-containing area and correcting for the size of each area. The size-distribution of DNA in each fraction was estimated by drawing a line through the ladder and extrapolating to other parts of the gel (Supplementary Figure S1D and E). Because ssDNA fragments are placed directly above dsDNA of the same size, the size-distribution of ssDNA could be estimated by using the coordination of bands in the ladder. Estimation of the size-distribution of undigested DNA, that did not migrate into the gel efficiently, could not be achieved as that fraction includes large DNA molecules of various sizes that are not resolved in the gel. Statistical analysis of repair experiments was done with Student’s *t*-test.

### Alkaline comet assay

Formation of interstrand crosslinks was analyzed with the alkaline comet assay (30,31). Confluent BJ cell cultures in T25 flasks were treated with 5 μg/ml cisplatin in serum free medium or 0.5–5 μg/ml MMC for 24 h in media containing 10% serum. At the end of treatment, medium was removed and cells were further treated with PBS containing 100 μM hydrogen peroxide (H_2_O_2_) for 15 min at 37°C to induce formation of DNA breaks (30). Cells were then trypsinized with 0.05% trypsin, PBS was added for total volume of 1 ml, and the cells were counted using Countess Automated Cell Counter (Life Technologies) after trypan blue staining. Cells were centrifuged at 1000 rpm for 1 min and resuspended in ice-cold PBS at 1 × 10^5^ cells/ml. The alkaline comet assay was then carried out using a CometAssay Kit (Trevigen, Gaithersburg, MD, USA cat no. 3950-075-02) according to the manufacturer’s instructions. Cells were mixed with molten 1% LM Agarose (1:10 cell suspension to agarose ratio) (Trevigen, cat. no. 4250-050-02) and 50 μl of the mixture were placed on the slide. The gel was allowed to solidify at 4°C for 10 min. The slides were first incubated with lysis solution on ice for 1 h and then in alkali unwinding solution (pH>13, 300 mM NaOH, 1 mM EDTA) at room temperature for 30 min. Alkaline comet assay electrophoresis was carried out in alkaline electrophoresis solution (pH >13, 300 mM NaOH, 1 mM EDTA) at 1 Volt/cm and 300 mA, with the slides placed equidistant from the electrodes in a Wide Mini-Sub Cell GT (BioRad) at 4°C for 40 min. After electrophoresis, the slides were washed two times in 100 ml H_2_O for 5 min, once in 100 ml 70% ethanol and then allowed to air dry at 37°C for 15 min. The DNA was stained with 1× SYBR Green I (Molecular Probes, Eugene, OR, USA) in TE buffer (10 mM Tris, 1 mM EDTA, pH 7.5) and visualized with LSM 5 Pascal (Zeiss) fluorescent confocal microscope. The Comet images were analyzed using ImageJ software (NIH) for tail moment (fraction of DNA in the tail × tail length), and 100 comets were scored in triplicate experiments for each sample. Control cells CC0–CC3 (Trevigen cat no. 4256-010-CC) with standardized DNA damage were run with the samples.

### Adaptor ligation

Adaptors N Bam 12 (5′-GATCCTCCCTCG-3′) and N Bam 24 (5′-AGGCAACTGTGCTATCCGAGGGAG-3′) (32) were ligated to Mbo I-digested human genomic DNA. The reaction included 100 pmols of each primer, 1 μg DNA template, 3 μl ligation buffer (Fermentas) and 1,3 mM MgCl_2_ in a final volume of 28 μl. The sample was heated to 55°C and the temperature lowered to 4°C at the rate of 1°C/min. The sample was put on ice and 800 U of T4 DNA ligase (Fermentas) added at 4°C for a final volume of 30 μl. The ligation reaction was at 15°C for 16 h. After ligation, polymerase extension was carried out at 68°C for 10 min, with dNTP’s (0.2 mM), 4 μl 10 × polymerase buffer (Finnzymes) and 4 U Dynazyme polymerase (2 U/μl, Finnzymes) in a final volume of 40 μl.

### DNA elution, PCR and agarose electrophoresis

Each fraction of adaptor-ligated and cisplatin-treated DNA was characterized using ImageQuant 5.1 software. The image was printed out on an overhead and the gel cut according to separation lines. DNA from gel parts was subsequently eluted in 700 μl 0,1 × TLE in 1500 μl Eppendorf tubes at 4°C overnight at constant shaking. After elution, the liquid was removed from the gel and the sample was then concentrated to 70 μl using SpeedVac plus (Savant). Polymerase chain reaction (PCR) on DNA eluted from gel parts was subsequently carried out with primer N Bam24. The PCR included 4 μl of DNA eluted from the gel, 1 x PCR buffer (4 mM MgCl_2_, Idaho), 0.2 pmol/μl primer N Bam24, 0.2 mM each dNTP, 2.5 U KlenTaq1 polymerase (25 u/μl, AB Peptides) in a final volume of 20 μl. PCR was carried out in a Veriti 96 Well Thermal Cycler (Applied Biosystems) and consisted of denaturation at 94°C for 3 min, followed by 30 cycles of 94°C for 30 s, 60°C 45 s and 68°C for 1 min, followed by elongation at 68°C for 7 min. PCR products were analyzed with electrophoresis in 1.7% agarose. Apoptosis ladder detection was carried out with 4 μg DNA on 2% agarose gels at 70 V for 90 min.

## RESULTS

### Detection of crosslinks on NLA

**Figure 2.**
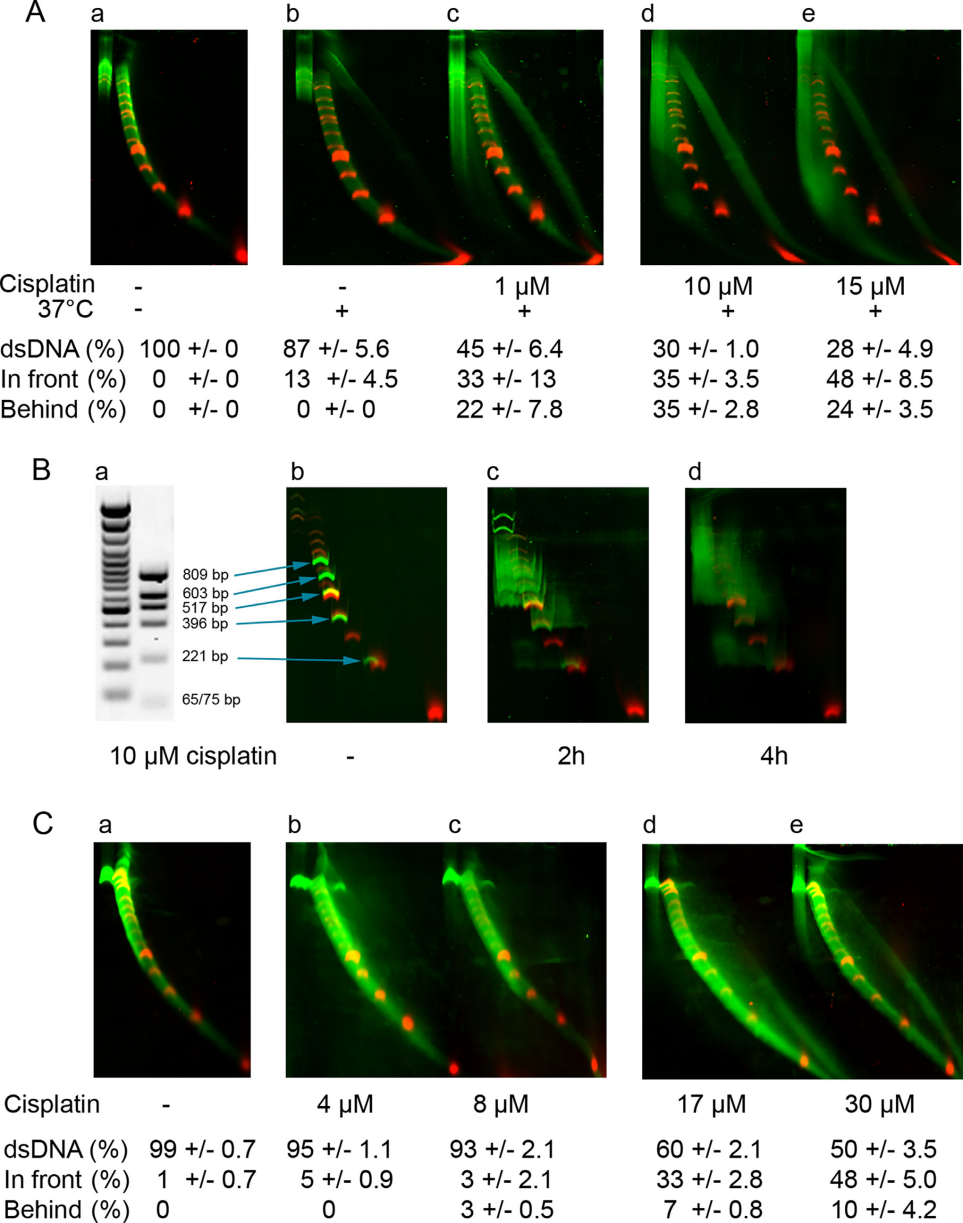
Cisplatin crosslinks in DNA detected with NLA. (**A**) Analysis of DNA crosslinks in a complex DNA sample. (a) Untreated but Mbo I-digested human genomic DNA (green smear) migrated with the double-stranded Cy5-labeled marker. (b) The same DNA after incubation at 37°C for 16 h showing slight heat-induced damage. (c–e) DNA samples treated with the indicated concentration of cisplatin. Quantification of fractions of duplicate experiments with standard deviations (SD) is shown. A significant increase in DNA migrating behind and in front of undamaged DNA was detected after treatment with all concentrations of cisplatin (*P* < 0.05). (**B**) Abnormal migration of individual DNA molecules containing interstand crosslinks or adducts including intrastand crosslinks detected with NLA. (a) Agarose analysis of GeneRuler 100 bp (lane 1) and sample pUC18 double-digested with Hinf I and SSp I to generate individually recognized fragments (lane 2). (b) NLA of sample (green bands) and marker, (c and d) NLA of sample after treatment with 10 μM cisplatin for indicated time. (**C**) Detection of DNA interstrand and intrastrand crosslinks in GM3440 fibroblast cell culture treated with different concentrations of cisplatin. A significant increase in DNA migrating behind undamaged DNA was detected after treatment with 8 μM cisplatin (*P* < 0.05).

Cisplatin, a well known crosslinking agent (33), was used to demonstrate that NLA could detect crosslinks in complex DNA samples in solution (Figure 2 and Supplementary Figure S2). In the original DNA sample, only undamaged DNA was seen. After incubation for 16 h at 37°C without cisplatin, a small amount of ssDNA had formed, presumably representing heat-induced damage and denaturation. Lesions induced with cisplatin were easily detected (Figure 2A). DNA migrating behind normal DNA was observed indicating interstrand crosslinks (arc to left of the dsDNA marker). Intrastrand DNA crosslinks bend DNA molecules to a differing degree depending on the damaging agent (33–36). DNA that migrated in front of the dsDNA arc corresponded to DNA molecules that were bent. Formation of ssDNA presumably occurred because a high number of intrastrand crosslinks and monoadducts caused denaturation of dsDNA. The DNA damage detected was dose-dependent (Supplementary Figure S2C).

The arc behind normal dsDNA could result from slower migration in the second dimension electrophoresis because of interstrand crosslinks, or relatively faster migration velocity of nucleic acids the first dimension, for example due to induction of the shorter A-helix DNA (37). To test this, a less complex sample with clearly identifiable and traceable bands was treated with cisplatin and analyzed with NLA (Figure 2B). Formation of interstrand DNA crosslinks caused the strands to remain covalently attached and migrate slower in the second dimension electrophoresis of NLA as seen, e.g. with the green bands migrating behind the 221 bp fragment. Fragments with intrastrand crosslinks migrated slower in the first dimension because of bending. After the second dimension they are placed in front of larger undamaged DNA fragments and vertically above the undamaged counterpart. This was also seen, e.g. with 221 bp DNA fragments placed in front of the 396 bp fragment. An alternative explanation would be that the band in front of the 396 bp band results from nicking. This is unlikely since non-specific nicking generates a smear that fades out (see later) and cisplatin in general does not form nicks in purified DNA. With longer incubation the bands become blurred indicating increased number of lesions per molecules including presumably formation of molecules with both interstand and instrastrand crosslinks.

We tested if DNA crosslinks could be detected in cell cultures treated with crosslinking agents. GM3440 cells, human fibroblasts with normal DNA repair, were treated with different concentrations of crosslinking agents. Treatment with biologically relevant concentrations of cisplatin (38) for 8 h, ranging from 4 to 30 μM, caused formation of the same types of dose-dependent DNA lesions as previously detected in DNA in solution (Figure 2C).

### Confirmation of the migration behavior of DNA with interstrand crosslinks

**Figure 3.**
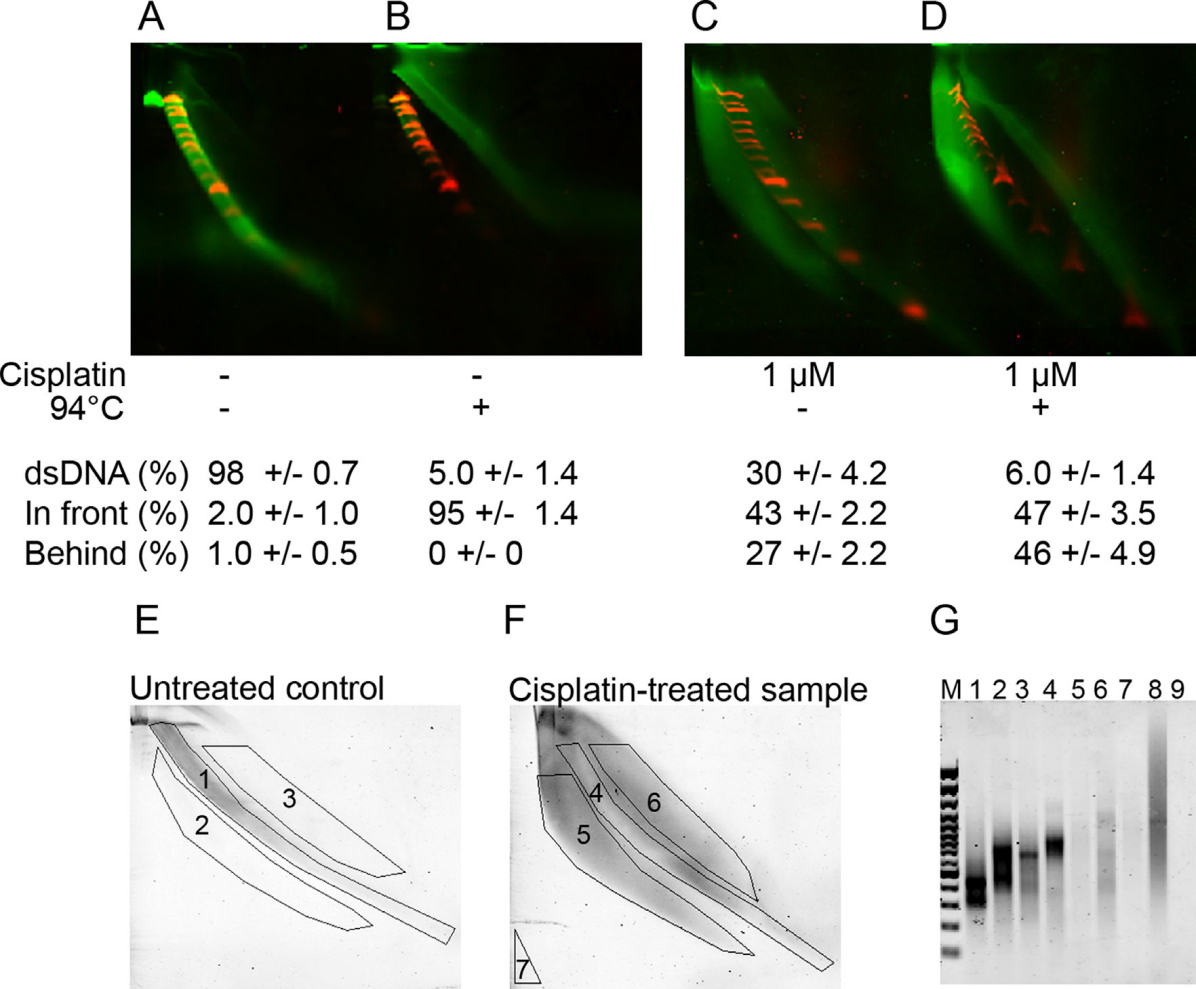
Confirmation that the DNA arc migrating behind normal dsDNA contained molecules with interstrand DNA crosslinks. (**A–D**) DNA in interstrand DNA arc was resistant to heat-denaturation. (A) The original undamaged MboI-digested human genomic DNA sample. (B) The original DNA after heat denaturation. (C) DNA after treatment with 1 μM cisplatin for 18 h. (D) DNA treated with cisplatin and heat denatured. (**E–G**) DNA in interstrand arc was refractory to amplification. Adaptor-ligated human genomic DNA (E) before and (F) after treatment with 1 μM cisplatin for 18 h was eluted from different labeled arcs of 2D-SDE gels. (G) PCR products of eluted DNA on an agarose gel. M: GeneRuler 100 bp Plus marker. Lanes 1–3 DNA amplified from the control (lane 1: dsDNA arc, lane 2: fraction behind dsDNA, lane 3: fraction in front of dsDNA). Lanes 4–6 DNA amplified from the cisplatin-treated sample (lane 4: dsDNA arc, lane 5: fraction behind dsDNA, lane 6: fraction in front of dsDNA. Lane 7: amplification in DNA-free gel, lane 8: positive control, adaptor-ligated DNA before gel-loading, lane 9: blank (H_2_O).

Two experiments were done to test if the arc migrating behind dsDNA was composed of DNA with interstrand crosslinks. The first was to heat denature a cisplatin-treated DNA sample before NLA. The original undamaged dsDNA sample that was heat treated prior to NLA migrated as ssDNA (Figure 3A and B). In solution, interstrand DNA crosslinks prevent full separation of DNA strands and cause quick renaturation of DNA. This caused DNA with interstrand crosslinks to migrate in the same manner as without heat treatment (Figure 3C and D). We also tested if DNA fractions in the gel were amplifiable by PCR. Adaptors were ligated to Mbo I-digested DNA sample before cisplatin-treatment, DNA from different arcs of the gel was isolated after NLA and the eluted DNA was amplified with the adaptors as primers. Neither strand of dsDNA containing interstrand crosslinks can serve as template. The PCR did not amplify products from DNA eluted from the gel part behind dsDNA, further confirming that the DNA migrating behind dsDNA contained interstrand DNA crosslinks (Figure 3E–G). In contrast, DNA was amplified from other parts of the gel. DNA migrating in front of dsDNA should contain intrastrand crosslinks on at least one strand. As both strands were retarded in the first dimension electrophoresis, the intact complementary strands also migrated in front of the dsDNA and were amplifiable (Figure 3G, lane 6). The amplified material in Figure 3G lane 2 presumably resulted from trailing fragments from the dsDNA arc. The large amount of non-amplifiable material in Figure 3G lane 5 presumably quenched amplification of corresponding dsDNA trailing elements in the cisplatin-treated DNA.

### Detection and repair of DNA crosslinks in FA cell lines with NLA

**Figure 4.**
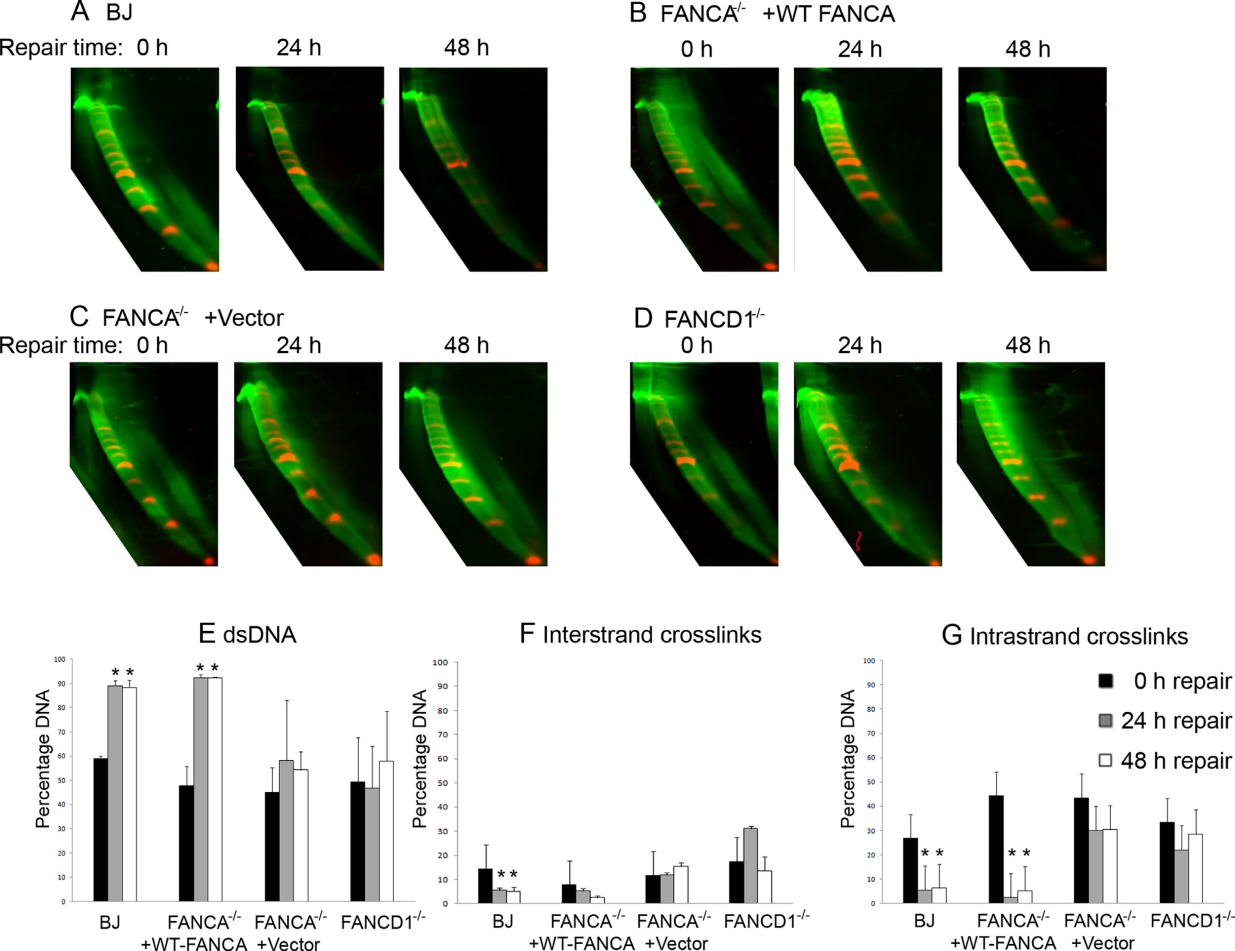
NLA of repair activity in fibroblast cell cultures. (**A–D**) BJ, *FANCA*^−/−^ +WT-*FANCA, FANCA*^−/−^ +Vector and *FANCD*1^−/−^ fibroblast cell line cultures. Quantitative measurements after repair are shown for (**E**) dsDNA, (**F**) interstrand DNA crosslinks and (**G**) intrastrand DNA crosslinks. The average percentage of each DNA fraction in triplicate experiments is shown, after no repair (black columns), 24 h repair (gray columns) and 48 h repair (white columns). Asterisks indicate statistically significant *P*-values.

We tested if NLA could be used to detect the different sensitivity of FA and wild-type cell cultures to MMC, a commonly used agent for the diagnosis of FA. The following cell lines were used: i) BJ, wild-type human fibroblasts; ii) *FANCA*^−/−^ +Vector, FA patient fibroblasts which have defective DNA crosslink repair due to biallelic mutations in the *FANCA* gene; iii) *FANCA*^−/−^ +WT-*FANCA*, the same FA fibroblasts transfected with a *FANCA* gene, which restores their DNA crosslink repair; iv) *FANCD1*^−/-^ FA patient fibroblasts which have defective DNA crosslink repair due to biallelic mutations in the *FANCD1/BRCA2* gene. Cytogenetic abnormalities detected after treatment with 50 nM MMC for 24 h correlated with cell types (Supplementary Figure S3 and Table S1). Fibroblast cultures were treated with 0.5 μg/ml and 5 μg/ml MMC. DNA was isolated immediately after MMC treatment ranging from 4–24 h. Damaged DNA detected by NLA was dose-dependent, but did not correlate with cytogenetic abnormalities. After treatment with 0.5 μg/ml MMC limited DNA crosslinks were detected in all cell types on NLA (Supplementary Figure S4). More DNA damage was detected after treatment with 5 μg/ml MMC (Supplementary Figure S4). The DNA damage detected included both interstrand and intrastrand DNA crosslinks at all time points. No differences in amount of DNA damage was detected between BJ, *FANCA*^−/−^ +WT-*FANCA* and the two repair deficient cell lines. Treatment for 6–8 h did not increase DNA damage compared to 4 h incubation. After MMC incubation for 24 h relatively more interstrand crosslinks were detected, along with accumulation of ssDNA in the repair deficient cell lines (compare Figure 4 to Supplementary Figure S4). The ssDNA may reflect accumulation of replication intermediates formed during the longer MMC incubation. DNA crosslinks were also detected after treatment of BJ cell cultures with different concentrations of DEB and melphalan (Supplementary Figure S5).

We tested if NLA could detect differences in repair of MMC damage between cells with wild type DNA repair capacity (BJ and *FANCA*^−/−^ +WT-*FANCA* cell lines) and cells with defective DNA crosslink repair (*FANCA*^−/−^ +Vector and *FANCD1*^−/−^ cell lines). Cell cultures were treated with 5 μg/ml MMC for 24 h and then allowed to recover in a MMC-free medium for 24 or 48 h (Figure 4A–D). After the indicated repair time an increase in the relative amount of dsDNA was detected in BJ cell lines (*P* < 0.05) (Figure 4A and E) and a decrease in interstrand crosslinks (*P* = 0.02 after 24 h repair) (Figure 4F) and DNA intrastrand crosslinks (*P* < 0.05) (Figure 4G). In the complemented *FANCA*^−/−^ +WT-*FANCA* cell line, an increase in dsDNA and decrease in intrastrand and interstrand crosslinks were also detected, but the decrease in the percentage of interstrand crosslinks was not statistically significant (*P* = 0.06 for 48 h repair) (Figure 4B and E–G). Consistent with impaired DNA crosslink repair, *FANCA*^−/−^ and *FANCD1*^−/−^ cells did not show a significant increase in the percentage of dsDNA, nor a significant decrease in either type of DNA crosslinks in the first 48 h post MMC exposure (Figure 4C and D). We also treated the cell cultures with 5 μg/ml MMC for 8 h and allowed them to recover in a MMC-free medium for 72–120 h so the cells would enter replication phase (Supplementary Figure S6). Effective repair of the interstrand crosslinks in the BJ and the *FANCA*^−/−^ +WT-*FANCA* cell lines was seen after 72 h repair, but not in the *FANCA*^−/-^ +Vector cell line. The DNA migrating in front of the dsDNA was removed in BJ and *FANCA*^−/−^ +WT-*FANCA* cell lines. In the *FANCA*^−/−^ +Vector cell line we observed increased DNA in front of the dsDNA after 120 h repair compared to 72 h repair, which could indicate accumulation of replication intermediates (Supplementary Figure S6).

### Comparison of NLA and the comet assay in detection of DNA crosslinks

**Figure 5.**
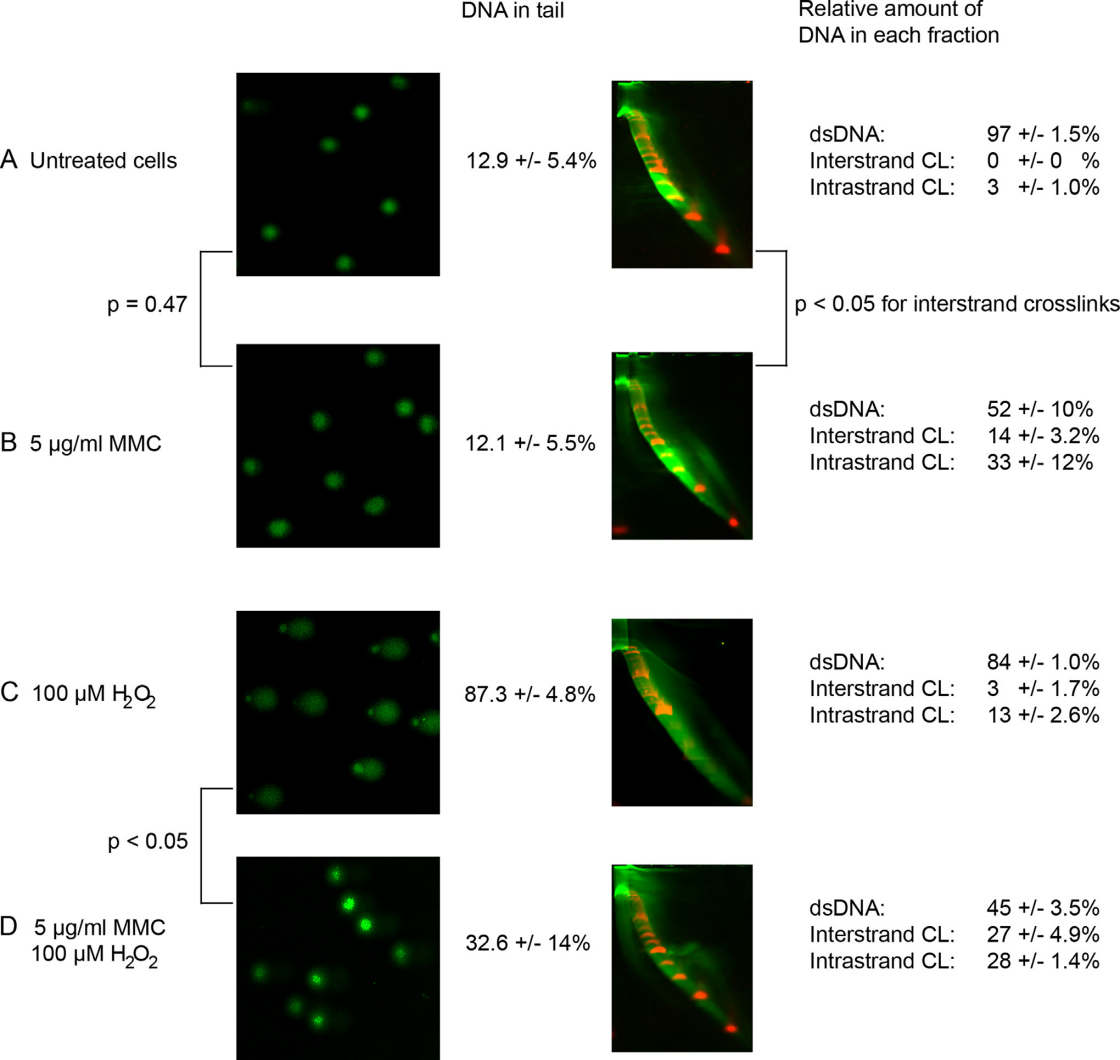
Comparison of NLA and the comet assay in detecting DNA crosslinks in cultured BJ cells. The score for the comet assay experiment is given in percentage of DNA in comet tails, for 100 scored comets in triplicate experiments. The difference in comet tail moment was not statistically relevant when (**A**)untreated cells were compared to (**B**) cells treated with MMC alone (*P* = 0.47), but relevant difference was detected between (**C**) H_2_O_2_-treated cells and (**D**) cells treated with both H_2_O_2_ and MMC (*P* < 0.05). The relative amount of interstrand and intrastrand DNA crosslinks for triplicate experiments analyzed with NLA are given. Increased formation of both types of crosslinks was detected when (**A**)untreated cells were compared to (**B**) cells treated with MMC alone (*P* < 0.05) and (**D**) after treatment with both H_2_O_2_ and MMC (*P* < 0.05).

The most common method currently used to detect DNA crosslinks is the comet assay. We compared the detection of DNA crosslinks with comet assay and NLA in BJ fibroblast cell cultures. The detection of crosslinks with the comet assay was based on the reduction in tail moment when cells treated with H_2_O_2_ alone were compared to cells treated with H_2_O_2_ and either cisplatin or MMC (30). DNA damage was not detected in an untreated control cell culture that did not receive treatment (Figure 5A). After treatment with MMC alone, a difference in migration was not detected on the comet assay, but both types of DNA crosslinks were detected with NLA (Figure 5B). H_2_O_2_ treatment resulted in increased DNA in tail in the comet assay (Figure 5C) and interstrand crosslinks were detected as decreased size of tail after treatment with both H_2_O_2_ and MMC (Figure 5D). In addition, ssDNA that resulted from oxidative DNA damage was detected with NLA after treatment with H_2_O_2_ alone (Figure 5C). Both types of DNA crosslinks were detected on NLA after treatment with H_2_O_2_ and MMC.

### Detection of DNA breaks on NLA

**Figure 6.**
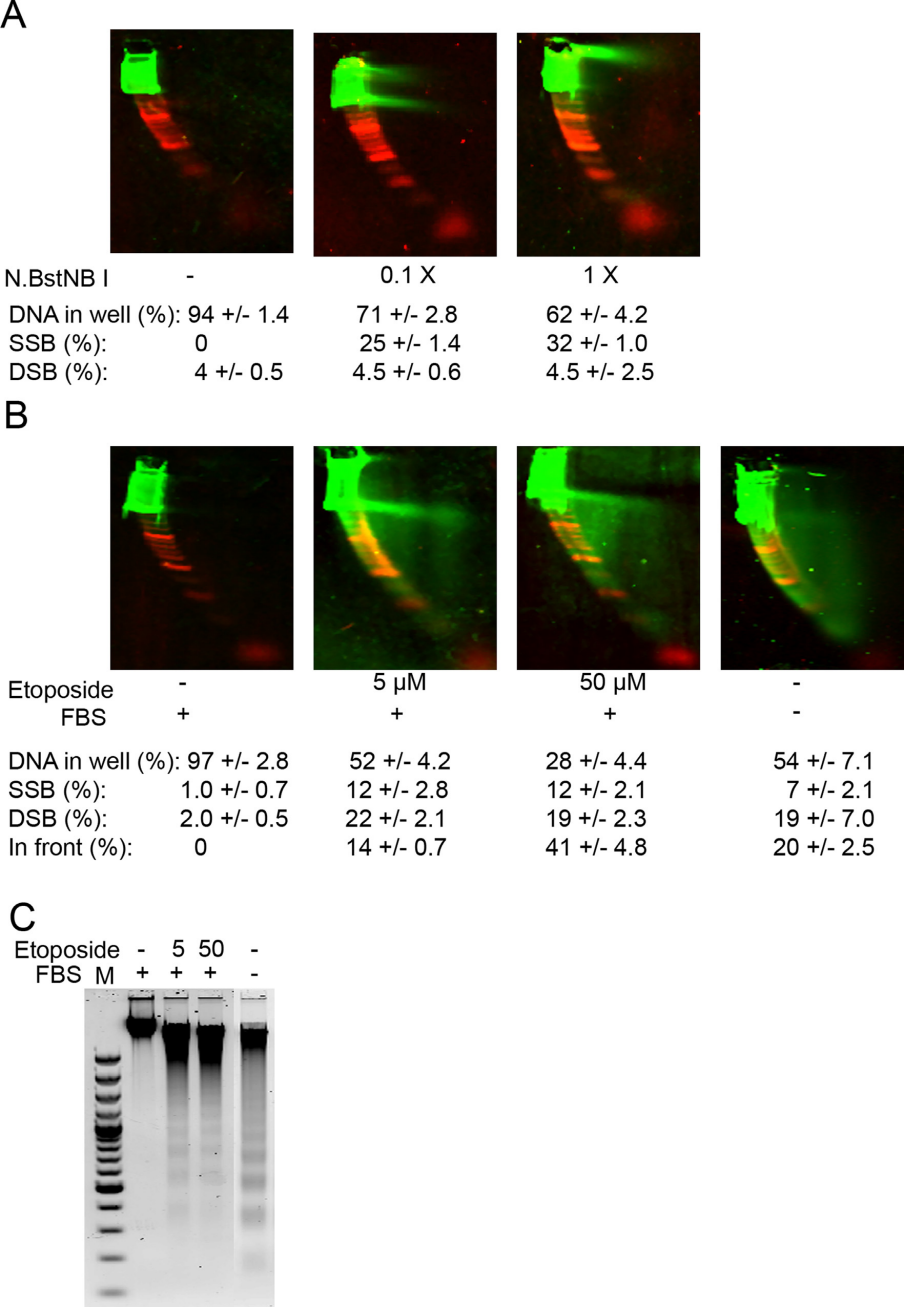
NLA of SSB and DSB in DNA in solution and in cell culture. (**A**) Undigested human genomic DNA sample treated with different concentrations of nicking enzyme N.BstNB I. (**B**) DNA from HUVEC cell cultures treated with etoposide or grown without FBS. (**C**) 2% agarose gels showing apoptosis ladder in HUVEC cells deprived of FBS or after treatment with etoposide. Quantification with SD is shown.

We induced formation of SSB and DSB by treating undigested human genomic DNA in solution with the site-specific nicking enzyme Nt.BstNB I, which has a 5 bp recognition sequence. The resulting SSB that were detected as a horizontal streak migrating from the DNA fraction that was too large to migrate into the gel (Figure 6A). Treatment of undigested human genomic DNA with the site-specific enzyme Nt.CviPII (3 bp recognition sequence) resulted in the same effect, albeit as expected at lower concentration of the enzyme since it is a more frequent cutter (data not shown).

We induced formation of SSB and DSB in HUVEC cell cultures using etoposide, a potent topoisomerase II inhibitor and inducer of apoptosis (12). We detected nucleosomal-sized double-stranded fragments of 180 and 360 bp (Figure 6B), that indicated an apoptosis ladder. From each nucleosomal-sized fragment we could detect a column that extended up to a horizontal streak extending from the fragments that were too large to enter the gel efficiently. The streak represented SSB. Of note there was increased density of material in the streak representing nucleosomal-sized bands or multiples thereof. For further confirmation that this pattern was due to apoptosis, the HUVEC cell cultures were starved of serum. A similar pattern of nucleosomal sized fragments and columns was detected (Figure 6B). The horizontal streak of SSB was less pronounced in the starved cells compared to etoposide-treated cells. Formation of apoptotic ladder in these samples was confirmed by analysis on agarose gels (Figure 6C).

**Figure 7.**
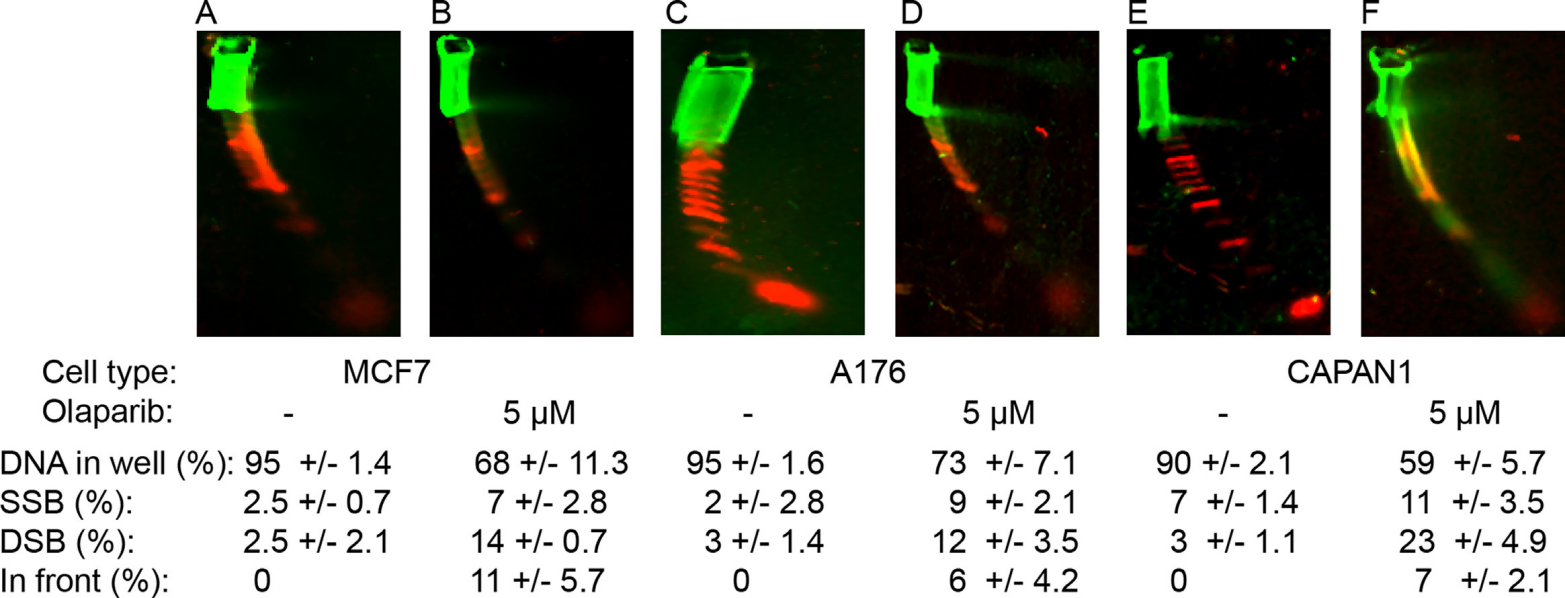
Detection of PARP1 inhibitor-induced breaks in cell cultures treated with olaparib. (**A**) Untreated MCF-7 cells (*BRCA2*^+/+^), (**B**) MCF-7 cells after treatment with olaparib, (**C**) untreated A176 cells (*BRCA2*^−/+^), (**D**) olaparib-treated A176 cells, (**E**) untreated Capan-1 cells (*BRCA2*^−/−^), (**F**) olaparib-treated Capan-1 cells. Quantification with SD is shown.

*BRCA2* ^−/−^ cells are deficient in homologous recombination (HR) and depend on non-homologous end-joining (NHEJ) pathways for DSB repair. PARP inhibitors cause SSB formation that is subsequently converted to DSB by the cellular replication machinery. In healthy replicating cells, DSB are repaired by the HR pathway, whereas *BRCA*^−/−^ cells lack effective DSB repair causing cell death. We tested if DNA breaks could be detected in cell cultures treated with the PARP inhibitor olaparib, a selective inhibitor of PARP1 that effectively inhibits repair of ssDNA breaks. MCF-7 cells, which are *BRCA2* wild type and the BRCA2 ± cell line A176 had increased SSB after treatment with olaparib (Figure 7A–D). The *BRCA2*^−/−^ cell line Capan-1 had substantially more DSB after olaparib treatment compared to the other two cell lines (Figure 7E and F).

## DISCUSSION

We describe comprehensive detection of multiple types of structural DNA lesions with NLA. The DNA lesions were detected by their different effects on the mobility of DNA molecules during NLA, resulting in the formation of distinctive migration patterns (Figure 1 and Supplementary Figure S7). Different types of lesions were detected simultaneously. The sensitivity of NLA appeared comparable to other methods for detection of interstrand DNA crosslinks (39), and topoisomerase-induced breaks (9).

We have adapted the NLA to a microgel format where the electrophoresis time is only 22 min (29). NLA is simple, robust and quantification of different DNA fractions in the sample is straightforward. Using the microgel format for NLA, as little as 5 ng sample of genomic DNA is needed for analysis. The NLA protocol, including DNA isolation and manipulation, can be completed in 5 h using a microgel, which is comparable to the time needed for the comet assay. While the comet assay can be carried out with fewer cells than NLA, a greater technical skill is needed for the comet assay. The cost of reagents and consumables for the two methods is similar.

Both NLA and the comet assay were able to detect DNA interstrand crosslinks (Figure 5), although several differences were observed between the two methods. Interstrand DNA crosslinks were detected via a modified comet assay in which cell nuclei were treated with a standard genotoxic agent to generate a tail of broken DNA fragments of certain size upon electrophoresis. A decrease in the tail moment indicates the presence of interstrand crosslinks that retard migration of DNA fragments (15,40). In contrast, NLA allows direct detection of DNA with crosslinks without using an extra genotoxic agent. It is therefore not influenced by variation in the effect of DNA-damaging agents, like H_2_O_2_ or ionizing radiation. The NLA method was able to analyze DNA molecules with both intra- and interstrand crosslinks along with damage causing DNA denaturation, while the comet assay could detect only DNA with interstrand crosslinks. The separation of DNA fractions with different types of DNA damage allows the researcher to isolate DNA from each fraction for further analysis of their composition. Importantly, DNA recovered from different arcs of NLA can be analyzed in order to test if specific motifs or genomic regions are more prone to form crosslinks. *In vitro* experiments have for instance revealed that methylated CpG sites are preferentially crosslinked with MMC (41).

DNA breaks can also be detected on NLA (Figure 1 and Supplementary Figure S7). Therefore, NLA can detect apoptosis ladders in undigested DNA samples as dsDNA nucleosomal-sized fragments migrating with the dsDNA marker (Figure 6). Of interest, besides the classical apoptosis ladder formed by CAD endonuclease, NLA can detect long fragments with SSB possibly generated with parthanatos and endoG nuclease, respectively (42,43). In addition, we detect vertical columns that extend upward from each nucleosomal-sized fragment into the horizontal streak resulting from SSB. The reason for the formation of the vertical columns is unclear, but they might reflect bending lesions retarded in the first dimension or more likely incomplete nicking in the DNA fragments as an intermediate in formation of the nucleosomal-sized fragments. Since single-stranded oligonucleotides can also be detected on NLA, the technique can be used to assess a much greater scope of stages of programmed cell death than is possible with a 1D agarose ladder. For example, the NLA apoptosis pattern after etoposide-treatment showed increased formation of SSB compared to the pattern for serum-starved cells (Figure 6), but this difference was not detected on agarose. This could indicate a difference in the programmed cell death caused by etoposide compared to serum starvation, as has previously been reported (44). In addition, we primarily detect formation of SSB and DSB when using NLA to analyze DNA from cells treated with the PARP1 inhibitor olaparib (Figure 7). The increased accumulation of DSB detected in the Capan-1 BRCA2^−/-^ compared to other cell lines is presumably due to their failure to repair strand breaks with HR.

Using NLA we found repair of MMC-induced DNA damage to be more effective in cell lines with normal DNA crosslink repair compared to DNA crosslink repair-deficient FA mutated cell lines, but both cell types contained similar numbers of induced DNA lesions prior to post-exposure repair. The repair of interstrand DNA crosslinks appeared to be less effective compared to the repair of intrastrand crosslinks. This may reflect that interstrand crosslinks are not fully repaired until the cell has started replication of the DNA (45). DNA migrating in front of normal DNA in MMC-treated FA mutated cell lines did not decrease after recovery in MMC-free medium (Figure 4C and D). In addition to intrastrand DNA crosslinks, other DNA molecules could potentially cause this type of NLA pattern. For example, replication intermediates would be detected by their migration in front of the normal DNA.

An important advantage of NLA is the ability to analyze DNA damage in cell-free systems (Figures 2 and 6). This would, e.g. allow analysis of DNA damage in body fluids, including plasma, saliva and urine, which is not possible with the comet assay. The sensitivity of the microgel system is adequate for analysis of DNA lesions in body fluids that may reflect disease state or response to therapy. Of particular interest would be studying the effects of radiation and chemotherapy and to test if NLA could be a possible method to test for ‘BRCAness’ (46). NLA analysis of purified DNA also has important applications in molecular procedures where crosslinks are formed such as Chip-Seq (21). NLA allows easy testing of the quality of DNA samples in biobanks and for genotoxicity both in naked DNA as well as in biological systems. A concern in genotoxicity testing is the sensitivity of the method, as some other methods might be more sensitive with regards to low concentration of particular DNA damage inducing agents. However, no methods allow as comprehensive detection of different types of DNA damage at the same time. NLA might therefore be the method of choice for screening for various genotoxic effects.

## Supplementary Material

Supplementary Data
