# Supplementary material for: Northern lights assay: a versatile method for comprehensive detection of DNA damage
Source: Nucleic Acids Res. 2018 Jul 24;46(20):e118. doi: 10.1093/nar/gky645 (PMC6237810; doi:10.1093/nar/gky645)
Supplement: Supplementary Data [file gky645_supplemental_files.pdf]

Northern Lights Assay: A Versatile Method for Comprehensive Detection of DNA Damage

Bjarki Gudmundsson, Hans Guttormur Thormar, Albert Sigurdsson, Wendy Dankers, Margret Steinarsdottir, Stefan Thor Hermanowicz, Stefan Thorarinn Sigurdsson, David Olafsson, Anna Margret Halldorsdottir, Stephen Meyn, Jon Johannes Jonsson

Supplementary material

Supplementary figure legends

Supplementary figure 1. Example of quantification of DNA within each fraction of a damaged sample. (**A**) NLA of a mixture of differently treated human genomic DNA samples: a) 50 ng undigested DNA, b) 50 ng Mbo I-digested DNA (dsDNA), c) 25 ng denatured MboI-digested DNA (ssDNA), d) 50 ng MboI-digested DNA treated with 1 µM cisplatin for 18 h at 37℃, e) 25 ng undigested DNA treated with 0.1× Nt.BstNB I at 55℃ for 1 h. (**B**) Scan of the Cy5-labeled ladder alone, showing relevant band sizes in bp used to define the coordination of fractions of undamaged dsDNA. (**C**) Scan of the ribogreen-stained sample showing definition of DNA-containing fractions: 1. Undigested DNA (purple), 2. Mbo I-digested dsDNA (green), 3. DNA migrating in front of intact dsDNA (red), including ssDNA and bent DNA, 4. DNA migrating behind dsDNA fraction (blue), containing interstrand crosslinks, 5. Streak of DNA containing SSB (brown), 6. DNA-free fraction used for background correction (black). The relative amount of DNA in each fraction is shown in the table. (**D**) Graph showing peaks for bands in the ladder, ranging between 100 – 3000 bp. (**E**) Size-distribution analysis of DNA in the sample shown for dsDNA (green), interstrand crosslinks (blue) and DNA migrating in front of the dsDNA arc (red).

Supplementary figure 2. Crosslinks in DNA detected with NLA at low concentration of cisplatin. (**A-B**) Mbo I-digested human genomic DNA treated with the indicated concentration of cisplatin. Quantification of fractions of duplicate experiments with standard deviations is included, $p < 0.05$ for both types of DNA crosslinks when untreated DNA was compared to DNA treated with 0.1 and 0.5 µM cisplatin. (**C**) Cisplatin induced a dose-dependent increase of DNA crosslinks. The graph shows a relative decrease of normal undamaged DNA and a relative increase of both interstrand and intrastrand crosslinks after treatment with cisplatin. The graph includes analysis of samples shown in Fig. 2.

Supplementary figure 3. Cytogenetic analysis of chromosomes from BJ, *FANCA*$^{-/-}$ +WT-*FANCA*, *FANCA*$^{-/-}$ +Vector and *FANCD*1$^{-/-}$ cell cultures treated with MMC. Increased numbers of aberrations (gaps, breaks, radials) were found in *FANCA*$^{-/-}$ +Vector and *FANCD*1$^{-/-}$ cell lines compared to BJ and *FANCA*$^{-/-}$ +WT-*FANCA* cell lines.

Supplementary figure 4. Comparison of the sensitivity of BJ, *FANCA*$^{-/-}$ +WT-*FANCA*, *FANCA*$^{-/-}$ +Vector and *FANCD1*$^{-/-}$ cell cultures to treatment with MMC. The cell cultures were treated with MMC

for the indicated incubation time and the DNA analyzed with NLA. The relative amount of DNA in each fraction is shown.

Supplementary figure 5. Detection of DNA crosslinks in cell cultures treated with DEB and melphalan. (A) Untreated BJ cells, (B) and (C) NLA after treatment of BJ cell cultures with different concentration of DEB for 24 hours. (D) and (E) BJ cells after treatment with different concentrations of melphalan for 24 hours.

Supplementary figure 6. NLA analysis of DNA repair activity in healthy and Fanconi anemia fibroblasts after a short incubation with MMC and long repair time. Triplicates of wild-type BJ, *FANCA*$^{-/-}$ +WT-*FANCA* and *FANCA*$^{-/-}$ +Vector fibroblast cell line cultures were treated with 5 µg/mL MMC for 8 hours and then allowed to recover and repair DNA in MMC-free media. The repair time is indicated above each figure. After the repair phases, the relative amount of dsDNA had increased ($p < 0.05$), and interstrand DNA decreased ($p < 0.05$) for both the BJ and *FANCA*$^{-/-}$ +WT-*FANCA* cell lines. The relative amount of dsDNA (0 h repair:72 h repair: $p = 0.30$, and 0 h:120 h: $p = 0.25$) and interstrand crosslinked DNA (0 h:72 h: $p = 0.50$, and 0 h:120 h: $p = 0.25$) did not significantly change in for the *FANCA*$^{-/-}$ +Vector cell line. The figures are shown in grayscale in order to increase sensitivity for the detection of interstrand crosslinks.

Supplementary figure 7. Patterns of undamaged and damaged DNA in NLA. (A) Undigested undamaged dsDNA (green) is too large to migrate into the gel, and the digested DNA forms an arc migrating with a Cy5-labeled DNA marker. (B) Single-stranded DNA forms a diagonal line in front of the marker in after digestion. (C) DNA molecules that contain interstrand DNA crosslinks have essentially the same migration velocity as dsDNA in the first dimension electrophoresis, but the strands do not separate after denaturation. Due to urea in the polyacrylamide gel, the strands do not reanneal. Molecules that contain interstrand DNA crosslinks have slower migration velocity in the second dimension electrophoresis and form an arc behind the double-stranded DNA marker. (D) Lesions causing bending in DNA molecules, such as loops, intrastrand DNA crosslinks and bulky adducts, result in slower migration velocity in the first dimension electrophoresis compared to undamaged counterparts. In the digested sample these lesions therefore form a smear in front of the undamaged DNA marker. NLA is not able to distinguish between these similar types of DNA damage. (E) Single-stranded DNA breaks (nicks and gaps) give rise to smaller fragments after denaturation. In the undigested sample the smaller fragments form a streak extending form DNA that was too large to migrate in the first dimension electrophoresis. In the digested sample, the smaller fragments migrate in front of the marker, resulting in a smear. (F) Double-stranded DNA breaks give rise to fragments that are small enough to migrate with the dsDNA marker into the gel efficiently in the undigested sample. (G) Apoptosis pattern gives rise to nucleosomal-sized dsDNA bands and single-stranded DNA breaks, with columns extending from the bands up to the streak of breaks. In general, DNA bands are present

in the streak corresponding to the size of nucleosomal fragments. This pattern is analyzed in the undigested sample only.

Supplementary Figure 1.

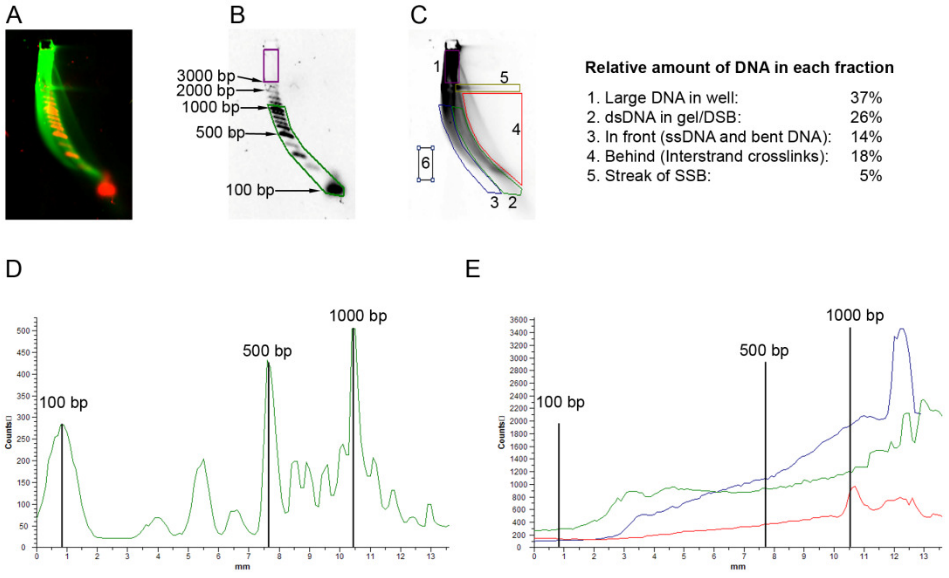

Supplementary Figure 2.

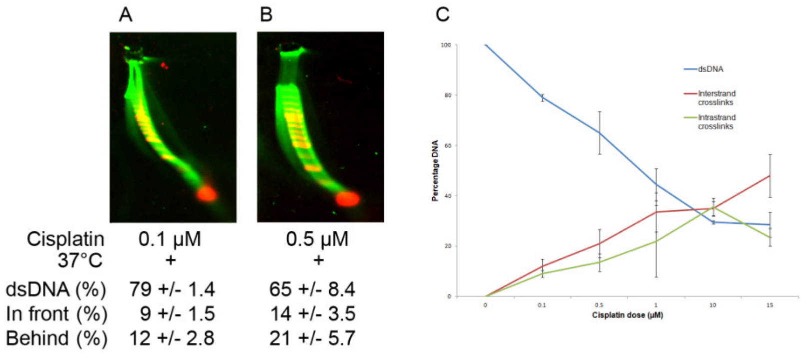

Supplementary Figure 3.

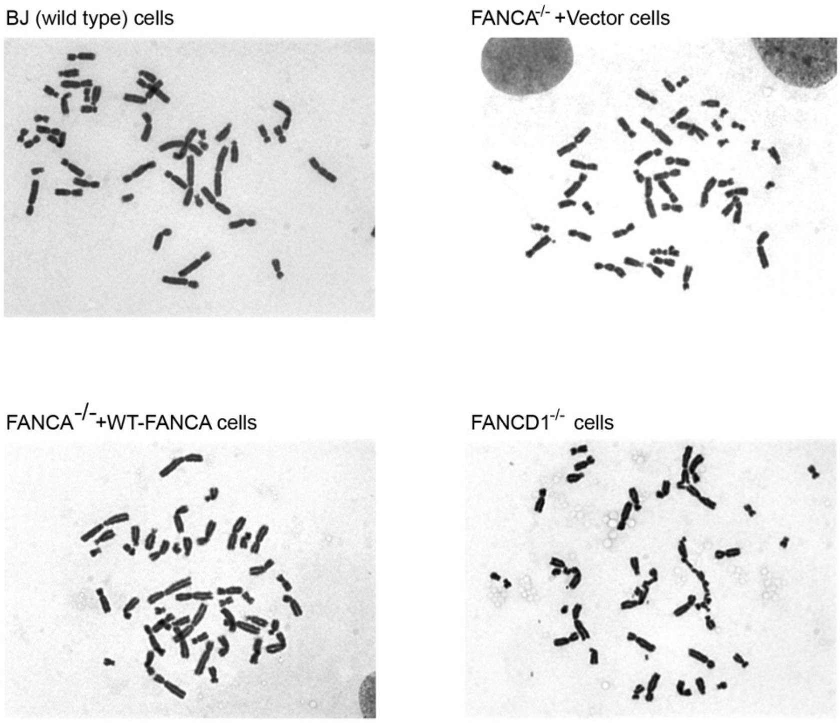

Supplementary Figure 4.

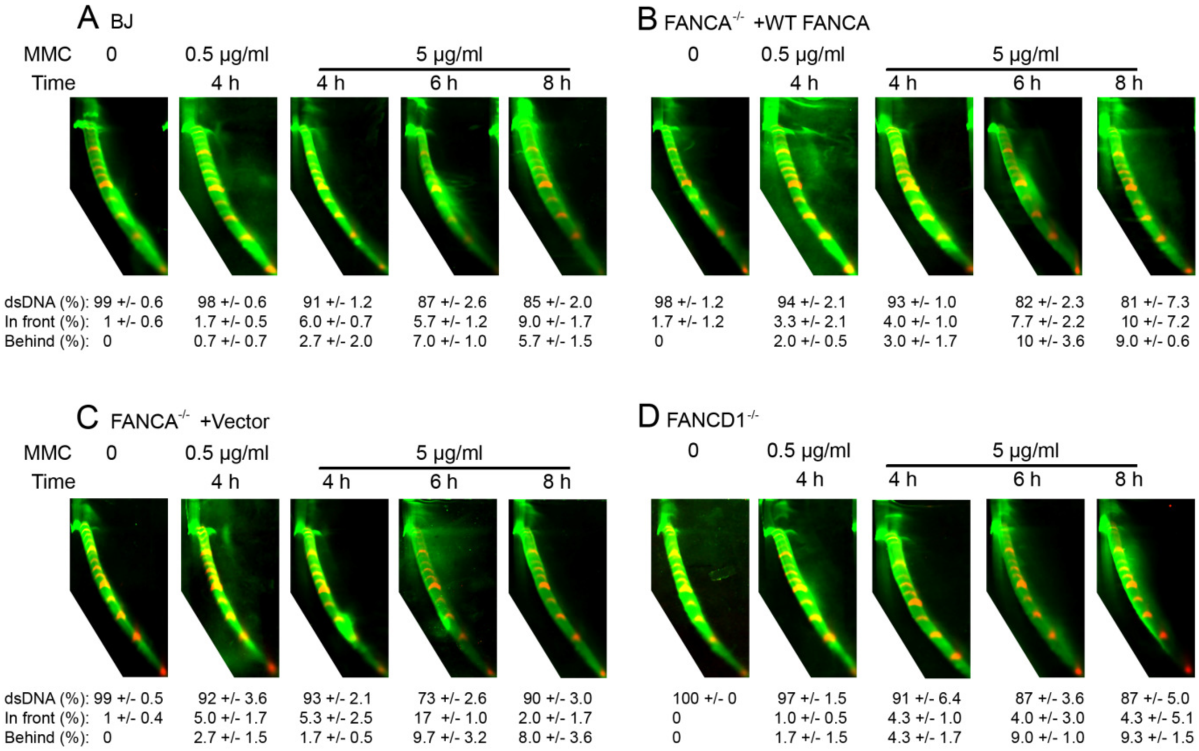

Supplementary Figure 5.

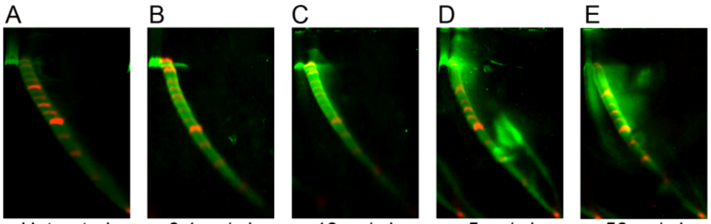

	Untreated cells	0.1 µg/ml DEB	10 µg/ml DEB	5 µg/ml melphalan	50 µg/ml melphalan
dsDNA (%):	98 +/- 1.0	97 +/- 1.4	92 +/- 2.1	59 +/- 6.3	42 +/- 2.2
In front (%):	2.0 +/- 0.7	3 +/- 1.4	5 +/- 1.2	39 +/- 6.3	40 +/- 2.2
Behind (%):	0	0	3,5 +/- 1.0	2.5 +/- 1.0	19 +/- 0.7

Supplementary Figure 6.

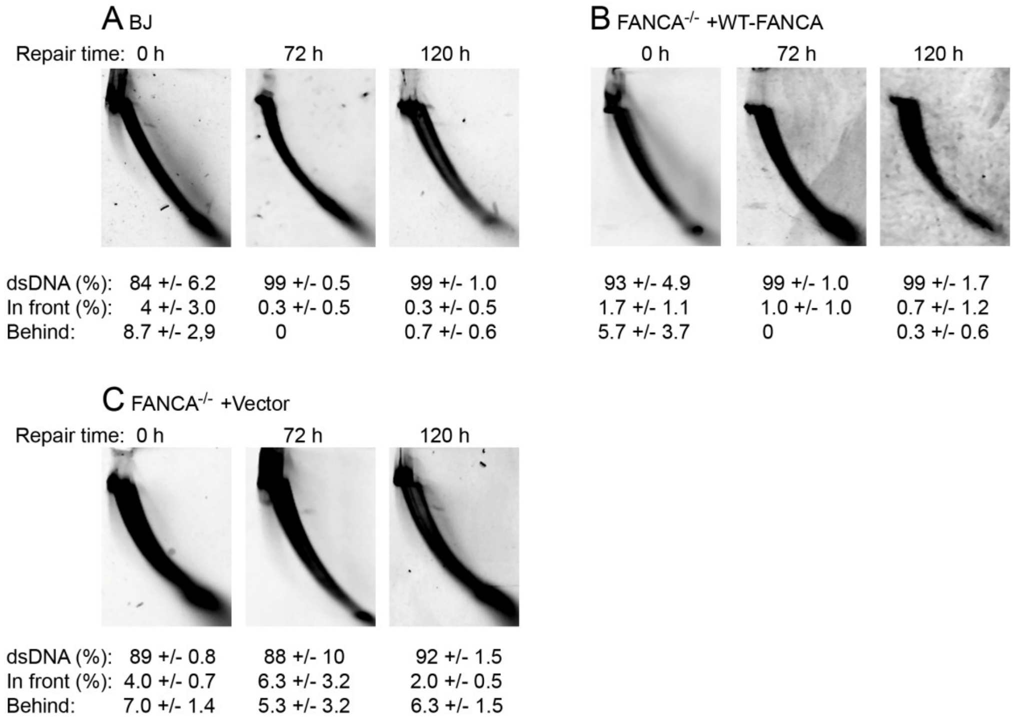

Supplementary Figure 7.

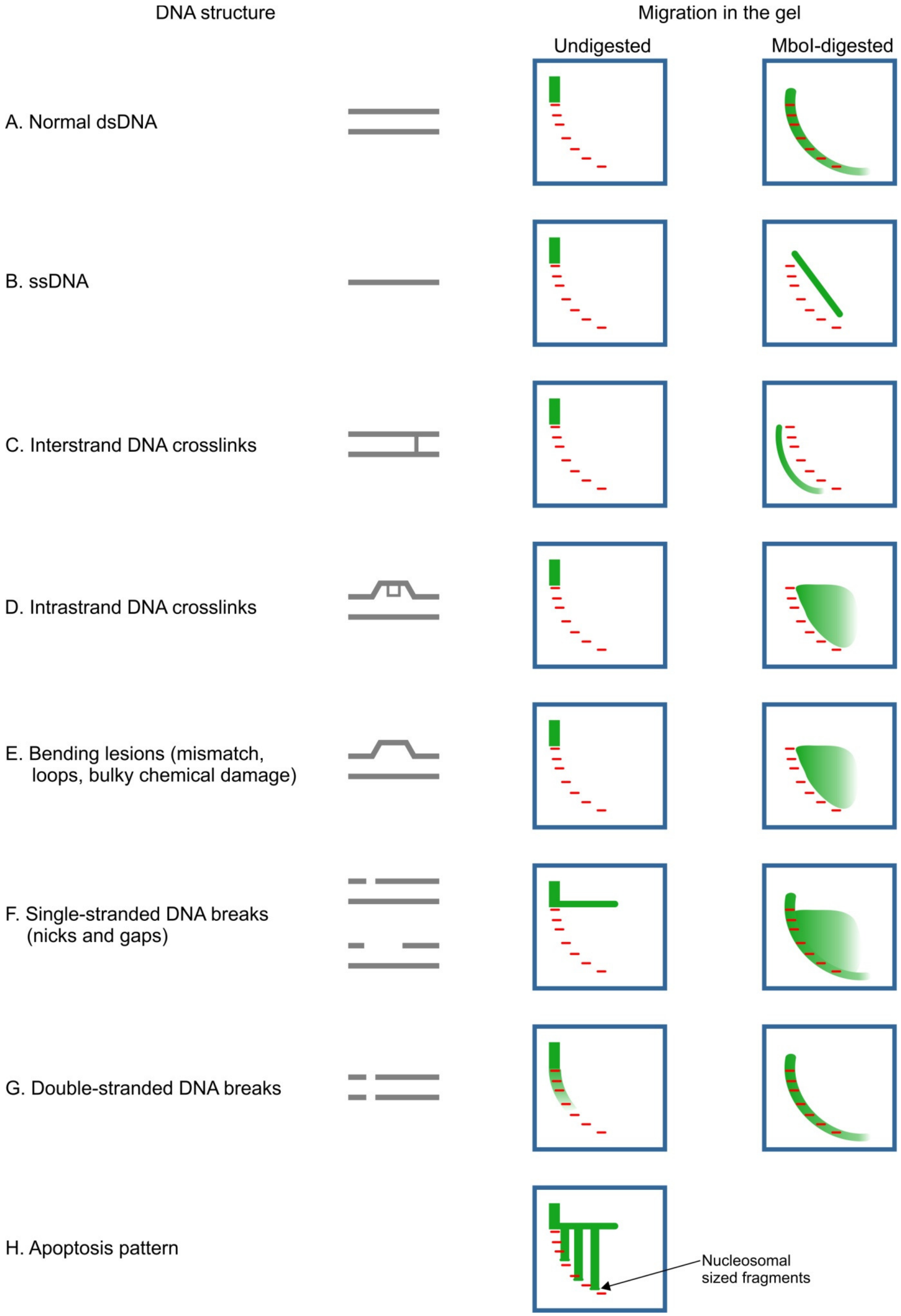

Supplementary Table 1. Cytogenetic analysis of MMC-treated cell cultures.

Cell type	No. of metaphases	No. with aberrations	No. of aberrations per cell
BJ (WT)	10	2	1-3
FANCA$^{-/-}$ + WT-FANCA	11	2	1-2
FANCA$^{-/-}$	17	16	1-10
FANCD1$^{-/-}$	12	12	1-20
